# Microstructural Influences on High Cycle Fatigue Crack Initiation Mechanism in Ti-Al-Mo-Cr-V-Nb-Zr-Sn Metastable β Titanium Alloy

**DOI:** 10.3390/ma18020336

**Published:** 2025-01-13

**Authors:** Chenxi Zhao, Yongxin Wang, Rui Hu, Guoqiang Shang, Yuxue Wu, Yunmei Lu

**Affiliations:** 1 State Key Laboratory of Solidification Processing, Northwestern Polytechnical University, No. 127, Youyi Road (West), Xi’an 710072, China; zhaochenxi@mail.nwpu.edu.cn (C.Z.); rhu@nwpu.edu.cn (R.H.); wyx0214@mail.nwpu.edu.cn (Y.W.); luyunmei@mail.nwpu.edu.cn (Y.L.); 2 Key Laboratory of Advanced Titanium Alloys, AECC Beijing Institute of Aeronautical Materials, Beijing 100095, China; shanggq1984@126.com

**Keywords:** high cycle fatigue, metastable β titanium alloy, crack initiation, microstructure, facet

## Abstract

In this work, the high cycle fatigue behavior and tensile properties of Ti-Al-Mo-Cr-V-Nb-Zr-Sn titanium alloy at room temperature with a basketweave structure and bimodal structure were studied. The results show that the fatigue strength of the basketweave structure is higher, while the balance of strength and plasticity of the bimodal microstructure is better. However, the fatigue performance of the bimodal microstructure is unstable due to the bilinear phenomenon of the S-N curve. By fractographic analysis and the study of the crystal orientation, as well as the slip traces of the primary α grains and β matrix at the facets, it was found that the facets are formed on the {$10\bar{1}1$}<$11\bar{2}0$> slip system with the highest Schmid factor, and the microcracks grow along the {110}<111> slip system in the β grain, but the driving force of microcrack propagation may exceed the restriction of crystallographic orientation. Based on the conclusions above, the phenomenological models of the fatigue crack initiation mechanism of Ti-Al-Mo-Cr-V-Nb-Zr-Sn titanium alloy are established.

## 1. Introduction

Metastable β titanium alloys, with their high specific strength, excellent corrosion resistance, superior deformation properties, and notable fracture toughness, are indispensable in the aerospace, automotive, and medical device sectors [1,2,3,4,5,6,7]. In the aerospace industry, these alloys are typically employed in load-bearing components or fasteners such as bolts or other components requiring high weight reduction, high load-bearing capacity, and superior performance [8,9,10]. In these applications, components often face fatigue failure induced by high-frequency vibrations, which significantly limits their service life. In high-cycle-fatigue scenarios, the initiation stage of fatigue cracks accounts for most of the total lifespan [11]. Therefore, comprehending the mechanisms of fatigue crack initiation is critical for extending and predicting the fatigue life of these components.

The initiation mechanisms of fatigue cracks in these titanium alloys are profoundly influenced by their microstructure. Huang et al. [12] investigated the high-cycle-fatigue behavior of the lamellar and bimodal microstructure Ti-5Al-5Mo-5V-3Cr-1Zr (Ti-55531) alloy at room temperature. It was reported that microcracks predominantly nucleated at interfaces of primary α (α_p_) and β_trans_ (β transformation microstructure) or within α_p_ particles in the bimodal microstructure. In the lamellar microstructure, cracks were initiated at interfaces between α films at grain boundaries and prior β grains or at secondary α (α_s_) and residual β interfaces. Yue et al. [13] investigated the fatigue performance of equiaxed and lamellar microstructure in the Ti-20Zr-6.5Al-4V alloy, identifying α dislocation pile-ups at the α/β interface as the preferred crack initiation sites. Shi et al. [14] proposed that coarse lamellar α phases were the primary sites for fatigue crack initiation in the basketweave-microstructure Ti-5Al-5Mo-5V-1Cr-1Fe titanium alloy. In general, the fatigue crack initiation of the metastable β titanium alloy may be due to the cracking of the α/β phase interface [15], the fracture along the grain boundary [16], and the cleavage of the primary α [17,18]. In recent years, the application of additively manufactured titanium alloys has gradually gained traction. However, the unique microstructural characteristics and defects of additively manufactured titanium alloys, such as porosity and heterogeneity, adversely affect their performance and limit their broader use. Studies have explored heat treatment to improve the deformation performance of additively manufactured titanium alloys [19]. At the same time, investigating the relationship between microstructural defects and fatigue performance is equally important for enhancing their high fatigue strength [20]. Therefore, an in-depth investigation into the microscopic mechanisms of fatigue crack initiation in titanium alloys is crucial for optimizing their performance and extending their service life.

Furthermore, dislocation slip behavior also plays a critical role in fatigue crack nucleation in titanium alloys. Therefore, it is essential to study the slip initiation mechanism within grains and the mechanism of slip transmission across grain boundaries or phase boundaries during fatigue. Generally, slip preferentially initiates in the α phase, which has led to many studies focusing on the Schmid factor (SF) of the α phase. Wang et al. [16] investigated the high-cycle-fatigue behavior of the hierarchical nanostructure (HN) and the lamellar microstructure (LM), which indicated that prismatic slip was activated in the α_p_ phase under cyclic loading, while basal slip was activated in the grain boundary α phase. Plastic deformation in HN primarily occurred within the short rod-like α_p_ phase and GB α phase. In LM, the dislocation slip predominantly took place within the β matrix and accumulated at the interfaces between the α_s_ and β phase and at grain boundaries. Wu et al. [21] investigated the high cycle fatigue crack initiation mechanisms in bimodal Ti-5Al-7.5V alloy and discovered that facets forming at crack origins correlated with α_p_ grains exhibiting medium or high SF for basal slip. Preferential crack initiation sites include elongated α_p_ particles aligned for optimal basal slip, and clusters of equiaxed α_p_ particles, resulting in the development of near-basal facets. During the high cycle fatigue deformation of the Ti-55511 alloy, the prismatic <a> slip system in the α_p_ phases was preferentially activated, followed by the basal <a> and pyramidal <a> slip systems [22]. Liu et al. [23] thought that high-density geometrically necessary dislocations accumulated with basal or prismatic slips, and then sub-grain boundaries were formed in the α_p_ grains, which caused microcracks.

Ti-Al-Mo-Cr-V-Nb-Zr-Sn is a novel metastable β titanium alloy with ultra-high strength, capable of achieving an exceptional strength–plasticity–toughness balance through the adjustment of heat treatment processes. However, studies on its fatigue performance, particularly the fatigue crack initiation mechanism, remain limited. This work investigates the high-cycle-fatigue behavior and fracture morphology of the bimodal and basketweave-microstructure Ti-Al-Mo-Cr-V-Nb-Zr-Sn titanium alloy. Using Electron Backscatter Diffraction (EBSD), the initiation sites of microcracks were analyzed to elucidate the fatigue fracture mechanisms influenced by the microstructure. The results aim to provide a certain reference for the microstructure selection and anti-fatigue manufacturing of the Ti-Al-Mo-Cr-V-Nb-Zr-Sn titanium alloy.

## 2. Material and Methods

### 2.1. Microstructure of Materials

The material used in this study is a metastable β titanium alloy Ti-Al-Mo-Cr-V-Nb-Zr-Sn. The β transus temperature is about 848 °C. The titanium alloy was melted by a triple-vacuum consumable arc, followed by repeated forging to obtain a 210 mm diameter bar. After subsequent heat treatments outlined in Table 1, with an air cooling rate of 60 °C/min and water cooling rate of 400 °C/min, forging blanks with a thickness of 90–100 mm were obtained, exhibiting basketweave and bimodal structures.

**Figure 1:**
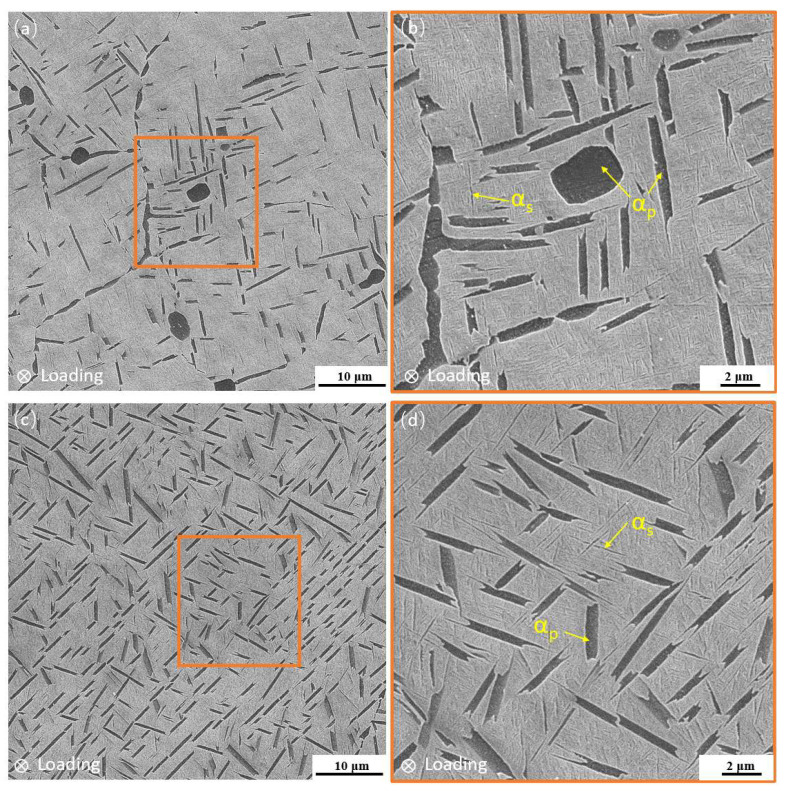
(**a**,**b**) The basketweave microstructure and (**c**,**d**) bimodal microstructure of Ti-Al-Mo-Cr-V-Nb-Zr-Sn titanium alloy.

The microstructures of the Ti-Al-Mo-Cr-V-Nb-Zr-Sn metastable β titanium alloy are shown in Figure 1, which are the secondary electron (SE) morphologies obtained by a scanning electron microscope. The basketweave structure is composed of coarse α laths and fine lamellar α phase dispersed in the transformed β phase. There is no original β grain boundary, and the α laths present an interlaced woven shape, as shown in Figure 1a,b. The volume fraction of the α laths is about 17%, and the thickness of the lamellar α phase is 35–60 nm. The bimodal microstructure is composed of a discontinuous primary α phase distributed on the β matrix and a dispersed lamellar α phase precipitated from the β phase during the aging process, and there is no obvious grain boundary, as shown in Figure 1c,d. The morphology of the primary α phase is equiaxed or short rod-like, and the volume fraction is about 12%. The thickness of the lamellar α phase is 20–50 nm, which is slightly lower than that of the basketweave structure.

### 2.2. Tensile and Fatigue Tests

The samples used for the tensile test and high cycle fatigue experiment were cut from the forged stock by the wire cutting method. To eliminate the impact of machining defects at the surface of the samples on the experiments, the final processing step is to polish the sample surface with sandpaper, which ensures uniform and low residual stress on the machined surface. The room-temperature tensile properties were tested according to the GB/T 228.1-2010 standard [24], using round bar specimens with a working section diameter of 5 mm. The tensile test was performed on an INSTRON 5887 tensile testing machine, manufactured by Instron (Shanghai) Test Equipment Trade Co., Ltd., Shanghai, China, at a strain rate of 0.001 s^−1^.

The axial stress fatigue tests were conducted according to the GB/T 3075-2008 standard [25], using standard hourglass-shaped specimens with a total length of 80 mm and a minimum cross-sectional diameter of 5 mm. The testing equipment was a QBG-100 high-frequency testing machine, manufactured by Changchun Qianbang Testing Equipment Co., Ltd., Changchun, China. The fatigue test was carried out at room temperature in an air atmosphere. The stress concentration factor Kt = 1. A sinusoidal wave with a frequency of 120 Hz and a stress ratio of 0.1 was applied. The fatigue test was stopped when the sample fractured or fatigue life reached 10^7^ cycles.

The fatigue strength at 10^7^ cycles with different microstructures was calculated using the staircase method. For the staircase method, 15 replicates were used for each microstructure. The S-N curves were generated by plotting the fatigue life data, with the unbroken samples assigned a lifetime of 10^7^ cycles.

### 2.3. Characterization Methods

**Figure 2:**
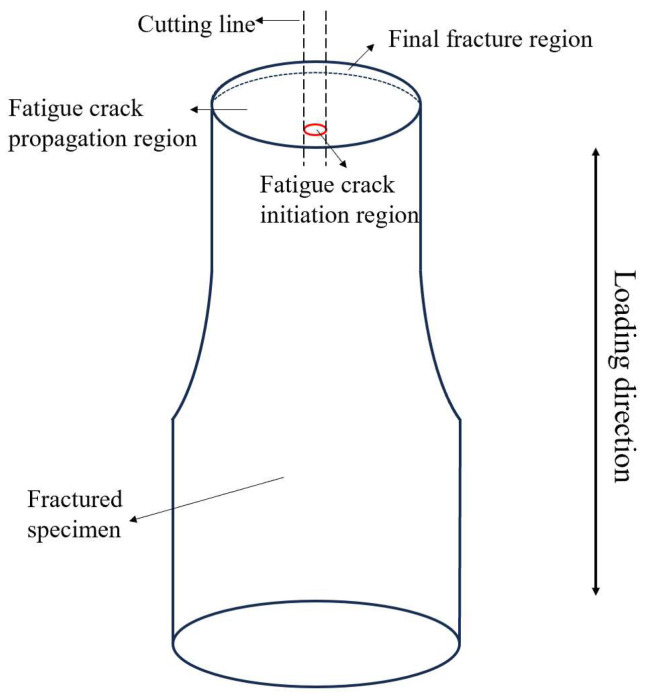
Schematic illustration of cutting lines on the fractured specimen.

The microstructure and the morphology of fatigue fractures were examined by TESCAN VEGA3 scanning electron microscope manufactured by TESCAN Co., Ltd., Shanghai, China, using SE mode with an accelerating voltage of 20 kV and a beam current of 10 nA. The fatigue samples were cut longitudinally by wire cutting to observe the microstructure below the fracture surface, as shown in Figure 2. Following mechanical polishing, the sample underwent an etching process using a corrosive solution with a ratio of V (HF):V (HNO_3_):V (H_2_O) = 10:7:83 to observe the microstructure. The samples for the EBSD analysis were mechanically polished and then subjected to 10 h of vibration polishing in the SiO_2_ suspension. The Channel 5 software package was used to analyze EBSD data.

## 3. Results and Discussion

### 3.1. Tensile Properties

**Figure 3:**
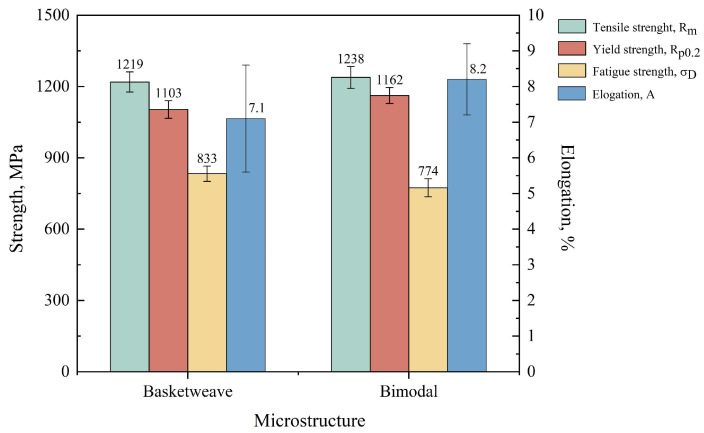
Mechanical properties of Ti-Al-Mo-Cr-V-Nb-Zr-Sn titanium alloy with different microstructures.

The tensile properties of Ti-Al-Mo-Cr-V-Nb-Zr-Sn titanium alloy with different microstructures are shown in Figure 3. The Ti-Al-Mo-Cr-V-Nb-Zr-Sn titanium alloy with bimodal microstructure has the best balance between strength and plasticity. The tensile strength is as high as 1238 ± 46 MPa, and the elongation reaches 8.2%. The tensile deformation of bimodal microstructure usually begins with the dislocation slip in the α phase [26]. With the increase in deformation, the slip occupies more and more α grains and expands to the surrounding transformed β phase. Since the spacing between slip bands is small, the stress concentration caused by dislocation pile-up at grain boundaries is not so severe, which delays the formation and development of voids. As a result, large deformation can be produced before the fracture of the alloy so as to obtain high plasticity. The strength and plasticity of the basketweave structure are lower than those of the bimodal structure, especially the reduction in area, which is only 50% of that of the bimodal structure.

However, the fatigue strength of the Ti-Al-Mo-Cr-V-Nb-Zr-Sn titanium alloy with the basketweave microstructure is higher, reaching 833 ± 32 MPa, while the fatigue strength of the bimodal microstructure is lower, which is only 774 ± 38 MPa. The fatigue ratio (σ_D_/R_p0.2_) of the alloy is an important index to verify the comprehensive fatigue performance [27]. The larger the fatigue ratio, the better the comprehensive fatigue performance of the alloy. The fatigue ratio of the Ti-Al-Mo-Cr-V-Nb-Zr-Sn titanium alloy with the basketweave microstructure is higher, which is 0.76, and that of the bimodal microstructure is slightly lower, which is 0.67. In general, the comprehensive fatigue performance of the basketweave microstructure is better.

### 3.2. Characteristics of the S-N Curve

**Figure 4:**
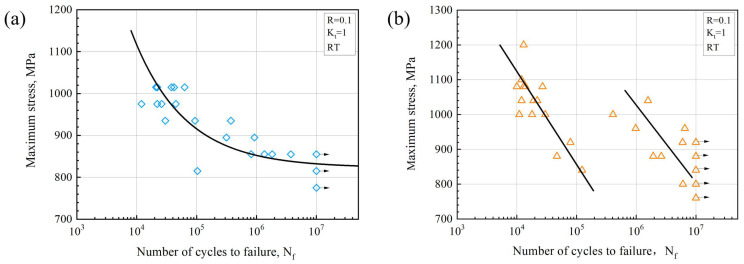
S-N diagrams of Ti-Al-Mo-Cr-V-Nb-Zr-Sn titanium alloy with the (**a**) basketweave microstructure and (**b**) bimodal microstructure.

The S-N curves of the Ti-Al-Mo-Cr-V-Nb-Zr-Sn titanium alloy with different microstructures are shown in Figure 4, which are obtained by the three-parameter power law. It is evident that the fatigue life of the basketweave-microstructure Ti-Al-Mo-Cr-V-Nb-Zr-Sn titanium alloy progressively extends as the stress decreases. Additionally, the S-N curve for the basketweave-microstructure Ti-Al-Mo-Cr-V-Nb-Zr-Sn titanium alloy indicates that the dispersion of fatigue life widens as the maximum stress decreases. When the stress level is relatively higher, the lifetime variability in fatigue is within one order of magnitude. When the stress level is 900 MPa, the fatigue life is two orders of magnitude different. However, the S-N curve of the bimodal-microstructure Ti-Al-Mo-Cr-V-Nb-Zr-Sn titanium alloy presents a bilinear relation. In each linear line, there is an increase in fatigue life as the stress decreases. The bilinear distribution represents the instability of the fatigue performance of the bimodal structure, which may be due to the randomness of the crack tip microstructure type (equiaxed/short-rod like α_p_). The dual nature of the fatigue S-N curve has also been observed in Ti-7333B [28] and Ti-10V-2Fe-3Al [29,30]. They believe that this is due to the unpredictability of surface and subsurface cracking.

### 3.3. Fractographic Analysis

One failure sample was selected from each of the two microstructures for fracture observation. The specific experimental parameters of selected samples are shown in Table 2.

**Figure 5:**
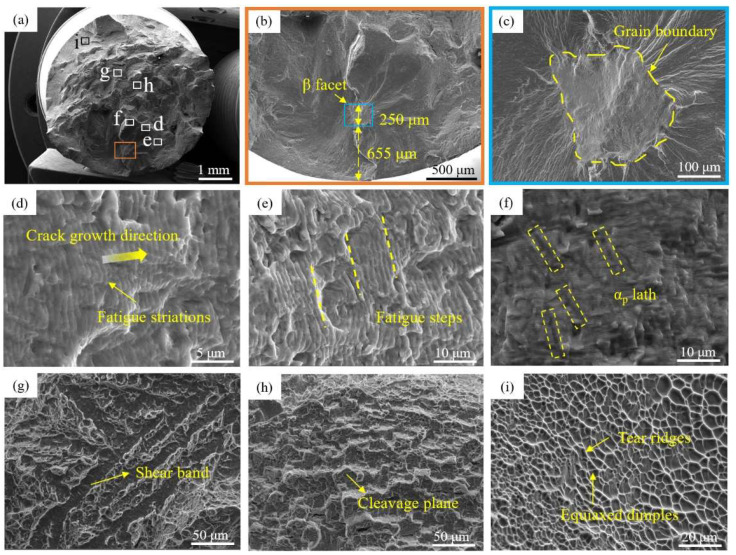
(**a**) Macroscopic fracture surface; (**b**,**c**) morphology of crack initiation site; (**d**) fatigue striations; (**e**) fatigue steps; (**f**) short rod-like α_p_ morphology appearing in the crack propagation zone; (**g**) shear band; (**h**) cleavage plane; (**i**) tear ridges and equiaxed dimples in the instantaneous break zone.

Figure 5 is the fatigue fracture morphology of the selected basketweave-microstructure Ti-Al-Mo-Cr-V-Nb-Zr-Sn titanium alloy specimen. Figure 5a shows the macroscopic morphology of the fracture surface. The fracture exhibits a notably rough surface. A lot of energy is absorbed in the process of crack propagation; so, it is difficult to break instantaneously, which is related to the excellent fracture toughness of the basketweave structure. Figure 5b,c displays the morphology of the fatigue crack origin. The images reveal that the source of the fatigue crack occurs just below the surface of the specimen, with the initiation point being a β facet located approximately 655 μm from the surface. The β facet is relatively flat, with a width of about 250 microns, and an obvious β grain boundary can be seen. Figure 5d displays the fatigue striations within the propagation zone, oriented perpendicularly to the direction of crack propagation. It is observed in Figure 5e that the fatigue striations are distributed step by step on planes of different heights. Under the action of cyclic stress, the cracks inside the material will gradually expand. However, due to the different microstructure characteristics of the materials, such as crystal orientation, grain boundary/phase boundary, inclusions and pores, the cracks will encounter different extents of obstacles during the expansion process. When the crack expands to a certain area with relatively large resistance, it may cause the shift in crack propagation direction to find a path with less resistance. This offset leads to a height difference in the crack front. Subsequently, the cracks propagate along their respective crystal planes, and the intersection of different fracture surfaces results in the formation of a step-like structure, known as fatigue steps. The α_p_ laths cut by the fatigue crack were also found in the fatigue striation zone (Figure 5f). In the rapid propagation zone, there are shear bands and cleavage planes. In the instantaneous fracture zone, tearing edges and relatively shallow equiaxed dimples can be observed.

**Figure 6:**
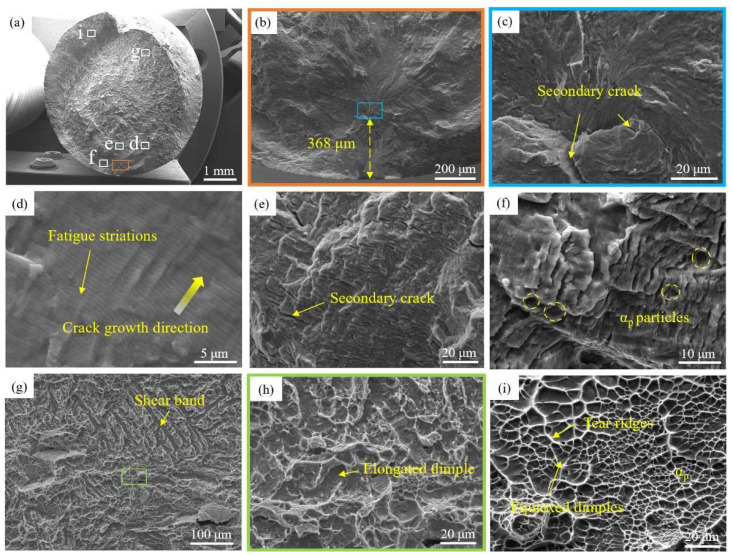
(**a**) Macroscopic fracture surface; (**b**,**c**) morphology of crack initiation site; (**d**) fatigue striations; (**e**) secondary crack in the fatigue crack propagation zone; (**f**) equiaxed α_p_ morphology appearing in the crack propagation zone; (**g**) shear band; (**h**) elongated dimple; (**i**) tear ridges and equiaxed dimples in the instantaneous break zone.

Figure 6 is the fatigue fracture morphology of the selected bimodal-microstructure Ti-Al-Mo-Cr-V-Nb-Zr-Sn titanium alloy specimen. Figure 6a is the macroscopic morphology of the fracture surface. Compared with that of the basketweave microstructure, the fracture surface is relatively flat. This is because, at the onset of fatigue stress loading, the rate of crack propagation near the fatigue initiation area is low. With continuous exposure to cyclic loads, the fatigue fracture surface becomes smooth due to the consistent opening and closing actions that cause it to be compressed and worn. The high-magnification figures of the fatigue source area are shown in Figure 6b,c. The fatigue crack has a single source and initiates inside the specimen. The fatigue crack initiation site is 368 μm away from the surface of the specimen and secondary cracks exist. Typical fatigue fracture characteristics such as fatigue striations (Figure 6d) and secondary cracks (Figure 6e) can be observed. The fatigue striations are more continuous and denser compared with those of the basketweave microstructure. There are traces of cracks cutting through equiaxed α_p_ particles at the fracture surface in Figure 6f. In the fatigue propagation zone, shear bands and elongated dimples can be observed, and the characteristics of a quasi-cleavage fracture can be observed. When it comes to the instantaneous fracture zone, the surface is composed of many interconnected dimples. The morphology is rough and shows the characteristics of a dimple fracture.

### 3.4. Crystal Orientation and Schmid Factor Analysis of α_p_

**Figure 7:**
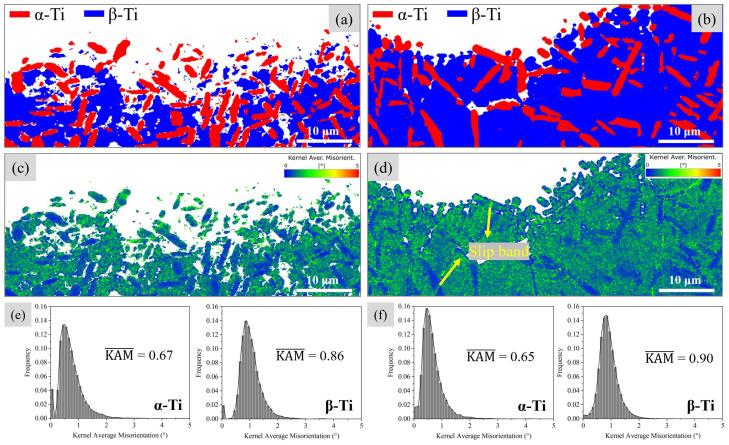
(**a**,**b**) The EBSD-derived phase maps of the basketweave and bimodal microstructure; (**c**,**d**) the corresponding KAM maps; (**e**,**f**) the corresponding local misorientation angle distribution of the α and β phases.

Kernel Average Misorientation (KAM) serves as an effective indicator for detecting local lattice distortions and qualitatively assessing deformation uniformity. Figure 7 shows the distribution of KAM values of the fatigue initiation region of the basketweave and bimodal microstructures. It is evident that the KAM values for the β phase are significantly higher than those for the α phase, suggesting that deformation predominantly occurs within the β phase. This occurs partly because the β matrix, with its body-centered cubic (BCC) structure, contains more slip systems than the α phase with a hexagonal close-packed (HCP) structure, making it more conducive to plastic deformation. This results in strain localization within the β phase due to its lower slip resistance. Additionally, research indicated that the precipitation of α_s_ from the matrix frequently causes lattice distortions and defects [31,32]. These defects hinder the movement of dislocations in α_s_, thereby enhancing its resistance to deformation.

**Figure 8:**
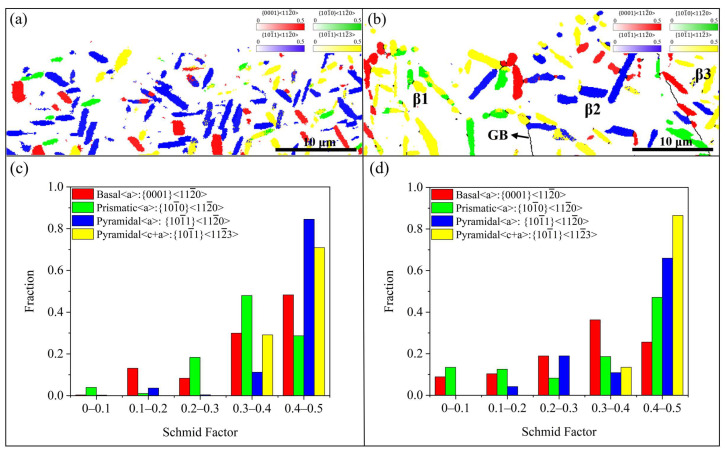
(**a**,**b**) Maps of the slip system with maximum SF of α_p_ in the fatigue crack initiation region of the basketweave microstructure and bimodal microstructure; (**c**,**d**) the numerical distribution of each slip system of α_p_ corresponds to each microstructure.

In metastable β titanium alloys, deformation and fracture usually start at the α phase, influenced by its higher elastic modulus relative to the β phase [33], along with its lower strength [34] and significantly reduced hardness, which is approximately one-third that of β phase [35]. Figure 8a,c shows the distribution of slip systems corresponding to the maximum SF of α_p_ in the fatigue initiation region. The basketweave microstructure predominantly activates the pyramidal <a> slip system with occasional engagement of the basal and pyramidal <c+a> systems. For the bimodal microstructure, the pyramidal <a> and pyramidal <c+a> slip systems are activated. In addition, the distribution of the slip system corresponding to the maximum SF is related to the β matrix. For the basketweave microstructure, the slip system of the maximum SF is uniformly distributed due to the same β grain in the region. However, for the bimodal microstructure, the region is composed of three β grains with different orientations; so, the slip system of maximum SF presents a regular distribution in the region: in the α phase of the β1 and β3 grains, the pyramidal <c+a> slip system is dominant, and in the β2 grain, the pyramidal <a> slip system is dominant. This non-uniform distribution may be caused by the influence of the orientation of β grains on the dislocation slip of α phase. The precipitation of the α phase from the β phase obeys the Burgers orientation relationship (BOR) [36,37]. Therefore, the orientation of the α particles will be affected by the orientation of the β grains, which, in turn, influences the activation of the α phase slip system. Due to the (semi-) coherent phase boundary formed according to BOR, the microstructure has lower interface energy and stronger stability. Meanwhile, the resistance of the dislocation slip is also reduced so that the two phases can coordinate deformation. The numerical distribution of each slip system is displayed in Figure 8b,d. For the basketweave microstructure, the pyramidal <a> and pyramidal <c+a> slip systems have the largest proportion in the high SF interval, which is up to 85% and 72%, respectively. Similar regularity is found in the bimodal microstructure, suggesting that the crystallographic orientation is favorable to the activation of the pyramidal <a> and pyramidal <c+a> slip systems.

The analysis reveals that both the pyramidal <a> and pyramidal <c+a> slip systems critically influence fatigue crack initiation in the Ti-Al-Mo-Cr-V-Nb-Zr-Sn titanium alloy. However, in addition to the SF, the critical resolved shear stress (CRSS) also plays a significant role in determining if the slip system is activated. Notably, the CRSS required to activate the pyramidal <c+a> slip system is much larger than that of the pyramidal <a> slip [38], with the basal slip system exhibiting the lowest CRSS of all. Hence, to further explore the microstructural features and crystallographic details of the crack initiation area, the crystal orientation of the α phase and the SF of the basal slip system and pyramidal <a> slip system in the crack source region of the two microstructures are studied.

**Figure 9:**
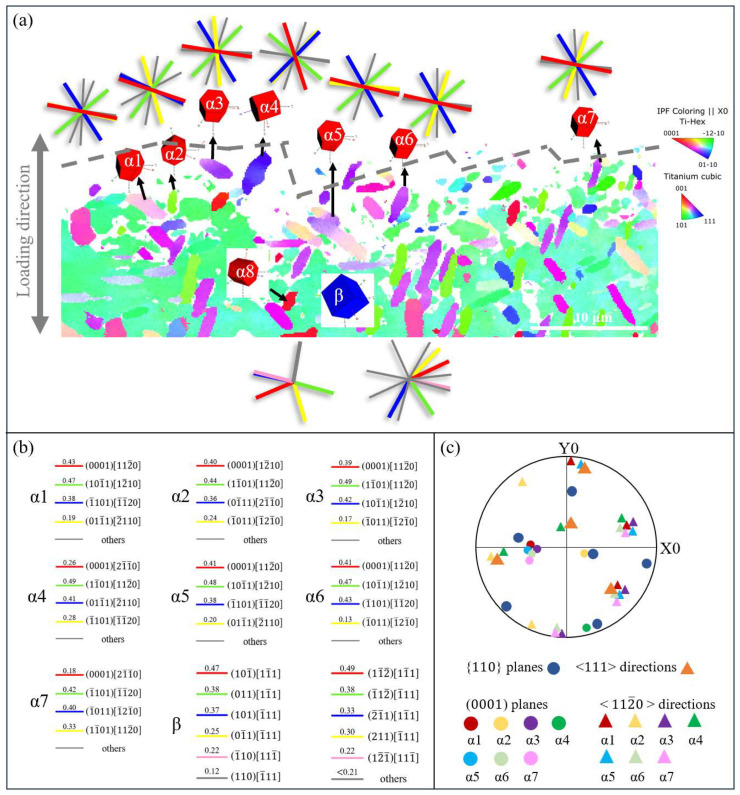
(**a**) IPF map of crack initiation region of basketweave microstructure; (**b**) SF for each slip system of pyramidal <a> slip system and pyramidal <c+a> slip system; (**c**) pole figure of selected α_p_ particles and β grain these belong to.

The fracture observation reveals that the fatigue crack of the basketweave microstructure initiates at a β facet. Figure 9 displays the Inverse pole figure (IPF) map of the area under the crack initiation region, where the crystal orientation and SF of seven selected α_p_ grains in the β grain at the fracture surface are studied. Figure 9a demonstrates that the microcrack propagation path within the initiation zone, which is marked as a gray dotted line, closely aligns with the cylindrical plane and is parallel to the slip traces of both the basal and pyramidal <a> slip systems. However, among all the α_p_ grains, the pyramidal <a> slip exhibits the highest SF, suggesting that the propagation of microcracks is oriented to the pyramidal plane. To be specific, the ($10\bar{1}1$) and ($1\bar{1}01$) plane dominates the activation of the slip system. SF calculations below the fracture surface reveal low values for the basal slip, prismatic slip, and pyramidal <a> slip in α8, recorded at 0.22, 0.02, and 0.12, respectively, classifying these as ‘hard’ grains less likely to initiate cracks. However, Huang et al. [39] reported that during deformation, certain α grains in the Ti-6Al alloy undergo rotation to accommodate larger plastic strains, which facilitates the softening of harder grains and promotes the activation of low-SF slip systems. Additionally, SF corresponding to the {110} and {112} slip planes of β grain are calculated, with the slip traces displayed in Figure 9a. It can be found that the direction of the β facet is approximately parallel to the {110} slip trace and the {112} slip trace with the highest SF. To be specific, it is the ($10\bar{1}$) <$1\bar{1}1$>slip system and the ($12\bar{1}$) <$\bar{1}11$> slip system both with an SF of 0.47, indicating that the β facet corresponds to the {110} and {112} plane with the highest SF. The pole figure shows that all selected α_p_ grains have a near Burgers orientation relationship (BOR) with the β grain.

**Figure 10:**
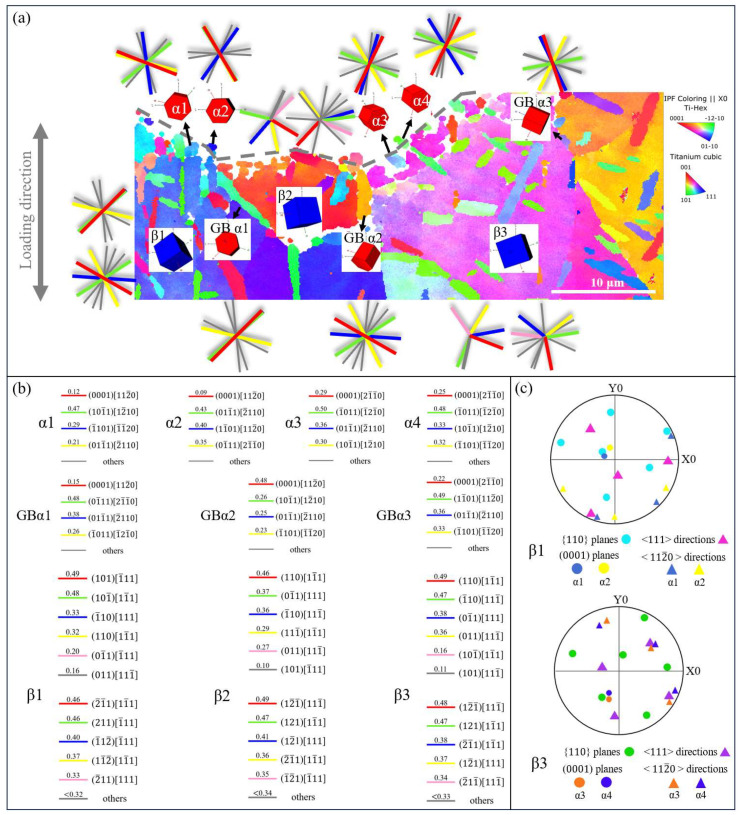
(**a**) IPF map of crack initiation region of bimodal microstructure; (**b**) SF for each slip system of pyramidal <a> slip system and pyramidal <c+a> slip system; (**c**) pole figures of selected α_p_ particles and β grain these belong to.

The IPF map of the crack initiation region of the bimodal microstructure and the slip trace with the corresponding SF are illustrated in Figure 10. The microcrack propagation path in the crack initiation region is close to the cylindrical plane, which has the same characteristics as the basketweave microstructure, and is parallel to the slip trace of the basal plane and the pyramidal plane. However, among all the four selected α_p_ particles, the SF of the pyramidal slip is the highest (0.47, 0.43, 0.5 and 0.48), suggesting that the propagation of the microcrack follows the pyramidal plane, particularly along the ($\bar{1}011$) slip plane. An analysis of the IPF diagram indicates that the fatigue crack initiation region comprises several β grains with varied orientations, interspersed with α_p_ grains at the grain boundaries. It has been pointed out that dislocation lines can be observed at GBα in the bimodal microstructure from TEM images, which affects the high cycle fatigue damage behavior of the alloy [40]. Therefore, the SF of several α_p_ at the grain boundaries below the fracture surface is calculated. The results show that the SF of the pyramidal slip system of most GBα is the highest, which is the same as that of other α_p_ particles at the fracture. However, one notable exception involves a high SF for the basal slip system at one GBα, indicating a change in GBα orientation that may affect the dislocation slip and promote crack nucleation through dislocation accumulation at phase boundaries. The SF and slip traces of the β grains are also shown in Figure 10a. The paths of microcrack propagation at β1 and β2 align closely with the {110} slip trace, possessing the highest SF, corresponding to (101) and (110) planes, respectively. However, the microcrack propagation path at β3 is not parallel to the slip plane, corresponding to the highest SF of any slip system. This implies that in the initial stage of microcrack propagation, the orientation of the β matrix has a great influence; that is, it propagates along the plane with the largest SF in the {110} slip system. Nevertheless, as the microcrack advances, the great driving force at the crack tip can exceed the influence of the β matrix’s orientation. In addition, the α2 particle does not strictly adhere to the BOR relationship, potentially due to the altered orientation of α_p_ resulting from deformation.

There are studies reporting that the formation of facets is mainly induced by basal or prismatic slip systems with a high SF [23,41]. However, insufficient activation of basal slip systems results in severe stress concentration and localized dislocation accumulation, and it can ultimately activate pyramidal <c+a> slip systems [42], consistent with the findings of this study. In addition, with increasing strain, slip systems characterized by a low SF can also be activated due to the combined influence of applied stress and internal stress, as noted in prior studies [43,44]. Li et al. [45] observed that in the Ti-5Al-2.5Sn alloy, certain low-SF slip systems become active under high-strain conditions. These low-SF slip systems (SF < 1.5) are predominantly initiated near grain boundaries or triple junctions, where significant local stress concentrations exist. Similarly, Zhang et al. [38] highlighted that deformation interactions between neighboring α grains could lead to load redistribution, thereby influencing the activation of slip systems.

### 3.5. Fatigue Crack Initiation Mechanisms

**Figure 11:**
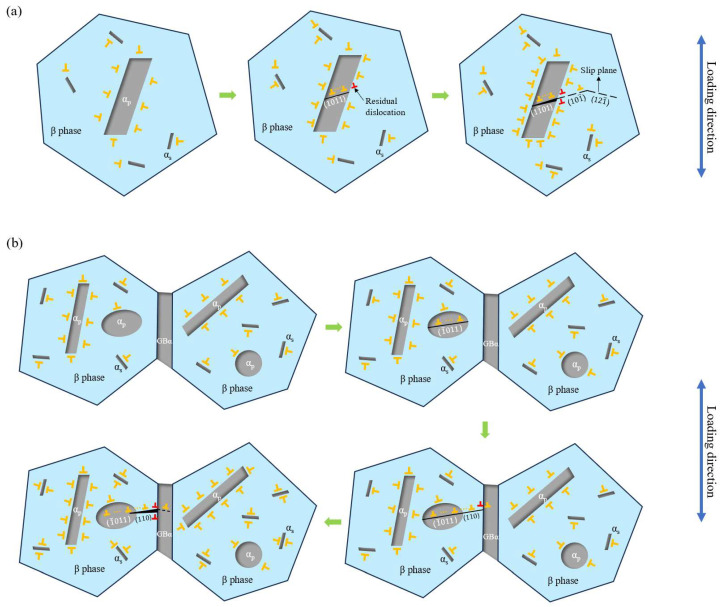
The phenomenological model for the fatigue crack initiation mechanisms of the (**a**) basketweave microstructure and (**b**) bimodal microstructure of the Ti-Al-Mo-Cr-V-Nb-Zr-Sn titanium alloy.

The phenomenological model to explain the formation of facets of Burgers-related colony microstructures has been developed, attributing facet formation to the accumulation of residual boundary dislocations caused by an interaction between the slip band and the grain boundary [46]. In addition, for homogeneous plastic deformation, the Von Mises criterion reveals that at least five active and independent slip systems are required [47]. However, the basal slip system and the pyramidal slip system can only provide two and four slip systems, respectively. Thus, the coordination of the β phase with various slip systems is required to achieve plastic deformation. As a result, the phenomenological model for the fatigue crack initiation mechanisms of the Ti-Al-Mo-Cr-V-Nb-Zr-Sn titanium alloy considering the dislocation slip between the α_p_ and β phases is established, as shown in Figure 11.

For both microstructures, at the initial stage of cyclic loading, defects such as dislocations are randomly distributed. Due to the incoherent or semi-coherent nature of the phase boundary, the dislocation density is initially higher. For the basketweave microstructure, as the number of loading cycles increases, there is a significant increase in the density of dislocations around the α_p_ particles, and the dislocations inside the α_p_ phase begin to accumulate along the ($10\bar{1}1$) slip band. As the number of cycles further increases, the dislocation density begins to increase and accumulates at the interface of the α_p_ and β matrix. A part of the dislocations pass through the phase interface, leaving the residual boundary dislocations to form microcracks under the continuous stress loading. The microcracks will continue to expand along the ($10\bar{1}$) or ($12\bar{1}$) slip band of the β grain, resulting in the initiation of microcracks and the formation of a β facet. For the bimodal microstructures, GBα is involved. During repeated loading, the dislocations in α_p_ are activated and gradually aggregate along the {$10\bar{1}1$} slip band. Specifically, in most cases, it is on the ($\bar{1}011$) plane with the highest SF. Dislocations pass through the α_p_/β interface and continue to slip in the β grain on the (110) slip plane. With the increase in the number of cycles, dislocations continue to move, leaving residual dislocations after passing through the GBα/β interface, and fatigue crack initiates when the residual dislocations accumulate to a certain extent.

In addition, since the metastable β titanium alloy will precipitate dispersed a_s_ particles through heat treatment, these fine a_s_ and β matrix phase interfaces will hinder dislocation slip; so, the strength of the alloy is markedly enhanced [48]. Moreover, it has been observed that some microstructural changes such as prismatic slip bands, strong slip bands and compression twins were found in the a_s_ plates, which promotes the initiation and growth of micro-voids or microcracks within fine α_s_ plates [12]. Therefore, the effect of the interaction between the a_s_ particles and the β matrix on fatigue crack initiation should also be studied in further studies.

## 4. Conclusions

This study investigates the mechanical and high cycle fatigue performance of the Ti-Al-Mo-Cr-V-Nb-Zr-Sn titanium alloy with basketweave and bimodal microstructures through tensile and fatigue tests. The fatigue crack initiation mechanisms are interpreted according to the fracture analysis and crystallographic information of the α_p_ particles. The main findings are summarized as follows:

1. The Ti-Al-Mo-Cr-V-Nb-Zr-Sn titanium alloy with a basketweave microstructure has a better high cycle fatigue performance than that with a bimodal microstructure. The bimodal microstructure shows greater fatigue life dispersion. The bimodal microstructure shows a bilinear phenomenon on the S-N curve due to the uncertainty of the microstructure of the crack tip.
2. Both the basketweave microstructure and the bimodal microstructure exhibit high KAM values. The degree of plastic deformation of the β matrix is greater than that of the α phase due to the higher KAM value. This inconsistency of plastic deformation of the phases will lead to the nucleation of fatigue cracks.
3. In both microstructures of the Ti-Al-Mo-Cr-V-Nb-Zr-Sn titanium alloy, the primary slip system activated is the pyramidal <a>. The microcrack initiates on the pyramidal plane with the highest SF of α_p_ in most cases. However, the propagation of microcracks can ignore the influence of crystallographic orientation due to the large driving force at the crack tip.
4. The phenomenological models of fatigue crack initiation in the basketweave and bimodal microstructures of the Ti-Al-Mo-Cr-V-Nb-Zr-Sn titanium alloy are established to explain the possible mechanisms of fatigue crack initiation.

## Figures and Tables

**Table 1:** Heat treatment process of the Ti-Al-Mo-Cr-V-Nb-Zr-Sn titanium alloy.

Microstructure	Solution-Treated T/Time	Cooling	Aging T/Time	Cooling
Basketweave	816 °C/120 min	Water cooling	535 °C/480 min	Air cooling
Bimodal	820 °C/120 min	Air cooling	540 °C/480 min	Air cooling

**Table 2:** Experimental parameters of fatigue tests of selected samples.

Microstructure	σ_max_ (MPa)	N_f_ (Cycle)	Location of Crack Source
Basketweave	940	1.355 × 10^6^	Sub-surface
Bimodal	800	6.056 × 10^6^	Sub-surface

## Data Availability

Due to privacy restrictions, we are currently unable to make the data publicly accessible. However, we would like to emphasize that these data can be made available upon reasonable request by contacting the corresponding author, provided that such requests comply with privacy-related guidelines.
